# How Do Human-Driven Vehicles Avoid Pedestrians in Interactive Environments? A Naturalistic Driving Study

**DOI:** 10.3390/s22207860

**Published:** 2022-10-16

**Authors:** Shulei Sun, Ziqiang Zhang, Zhiqi Zhang, Pengyi Deng, Kai Tian, Chongfeng Wei

**Affiliations:** 1 Key Laboratory of Automobile Measurement and Control & Safety, Xihua University, Chengdu 610039, China; 2 Engineering Research Center of Advanced Energy Saving Driving Technology, Ministry of Education, Chengdu 610031, China; 3 Institute for Transport Studies, University of Leeds, Leeds LS2 9JT, UK; 4 School of Mechanical and Aerospace Engineering, Queen’s University Belfast, Belfast BT9 5AG, UK

**Keywords:** pedestrian–vehicle interaction, naturalistic driving study, pedestrian crossing intention, driving behaviour, scenarios classification

## Abstract

One of the major challenges for autonomous vehicles (AVs) is how to drive in shared pedestrian environments. AVs cannot make their decisions and behaviour human-like or natural when they encounter pedestrians with different crossing intentions. The main reasons for this are the lack of natural driving data and the unclear rationale of the human-driven vehicle and pedestrian interaction. This paper aims to understand the underlying behaviour mechanisms using data of pedestrian–vehicle interactions from a naturalistic driving study (NDS). A naturalistic driving test platform was established to collect motion data of human-driven vehicles and pedestrians. A manual pedestrian intention judgment system was first developed to judge the pedestrian crossing intention at every moment in the interaction process. A total of 98 single pedestrian crossing events of interest were screened from 1274 pedestrian–vehicle interaction events under naturalistic driving conditions. Several performance metrics with quantitative data, including TTC, subjective judgment on pedestrian crossing intention (SJPCI), pedestrian position and crossing direction, and vehicle speed and deceleration were analyzed and applied to evaluate human-driven vehicles’ yielding behaviour towards pedestrians. The results show how vehicles avoid pedestrians in different interaction scenarios, which are classified based on vehicle deceleration. The behaviour and intention results are needed by future AVs, to enable AVs to avoid pedestrians more naturally, safely, and smoothly.

## 1. Introduction

The deployment of autonomous vehicles (AVs) is still facing many challenges, particularly their application in complex interactive environments, where different types of road users are included, e.g., pedestrians [1,2,3]. In such interactive environments, AVs not only need to make appropriate motion decisions but also need to consider other road users’ responses to AVs [4]. However, currently, AVs generally consider pedestrians as dynamic obstacles without intention and generally make simple braking decisions and actions to stop when AVs encounter pedestrians, especially in the scenarios of pedestrians crossing the street [5]. One of the reasons for the unnatural decision or behaviour is that the driver’s habits and pedestrian crossing intention in the pedestrian–vehicle interaction are not considered [1]. Moreover, the pedestrian–vehicle interaction rationale is unclear due to the lack of real-world interaction data [6]. Therefore, it becomes essential to carry out interaction experiments and behaviour mechanisms analysis to understand how human-driven vehicles avoid pedestrians in interactive environments.

The experiment methods for pedestrian–vehicle interaction behaviour mainly include driving simulator experiments with/without virtual reality (VR)-based devices [3,7,8,9,10], designed experiments in closed field scenes [11,12], and naturalistic driving study (NDS) in open roads scenes [13,14,15]. Although the NDS has the disadvantage of efficiency and security compared to the other two methods due to the accidental and uncontrollable interaction environments, it can reflect the real behaviour of road users, which cannot be affected by experiment conditions and related deviations [16]. Pedestrian and vehicle behaviour data acquisition, e.g., pedestrian position, can often be carried out through fixed videography, in-motion videography, or other sensors [17,18,19,20], and most current studies focus on predicting whether pedestrians cross the street based on these data [21,22,23]. It is known that the pedestrian crossing intention can change at any time, and the driver’s subjective judgement on pedestrian crossing intention (SJPCI) can affect vehicle deceleration behaviour directly. Therefore, it is necessary to obtain the crossing intention data at every moment in the whole process of pedestrian–vehicle interaction. However, the approach to acquiring quantitative data of the SJPCI of each moment, e.g., the percentage of crossing probability, and its affect on vehicle behaviour have not been studied in the existing literature.

Existing studies have identified several objective factors that may affect the interactive process between vehicles and pedestrians, e.g., time-to-collision (TTC), vehicle speed, distance from vehicle to pedestrian [24,25,26,27,28]. However, it is unreasonable to only use the above objective factors to study the interaction behaviour mechanism. Pedestrians’ behaviours and intentions are easy to change with high agility, and they can quickly change their direction of travel. For example, pedestrians can make a sharp 90° turn without reducing their speed [29] and can quickly switch from standing to walking [30]. Therefore, an interaction behaviour study must be carried out based on the objective behaviour data coupled with SJPCI. Furthermore, most previous interaction behaviour studies were carried out at intersections or on signalized roads [31,32,33,34], and the interaction behaviour can be affected by these complex scenarios. Hence, the pedestrian–vehicle interaction scenario of interest should exclude intersections, traffic signals, bumps, and group pedestrians to reduce the influence factors.

In this paper, NDS was used to collect motion data of human-driven vehicles and pedestrians. Additionally, a manual pedestrian intention judgment system was first developed to acquire the quantitative data of SJPCI at every moment. A new approach to the analysis and classification of the pedestrian–vehicle interaction behaviour based on several metrics, e.g., SJPCI, TTC, and vehicle deceleration, is carried out to understand how vehicles avoid pedestrians. The vehicle deceleration behaviour patterns encountering pedestrians in different scenarios will lead to a better understanding and development of advanced driving assistance systems (ADAS) for future AVs.

## 2. Methods

### 2.1. Participants

Ten participants aged between 22 and 47 years old (M = 27.4, S.D. = 7.42) were recruited, consisting of employees and students of Xihua University. They were asked to fill in questionnaires and consent forms to access the basic information of drivers, e.g., their age, driving mileage, and driving violation information. All the participants grew up in China and held valid driving licenses. They had normal vision or were corrected to normal. In the past three years, all of their driving mileage exceeded 20,000 km, and participants had not experienced any traffic accidents with pedestrians, ensuring that the participants had safe and good driving habits.

### 2.2. Apparatus

**Figure 1:**
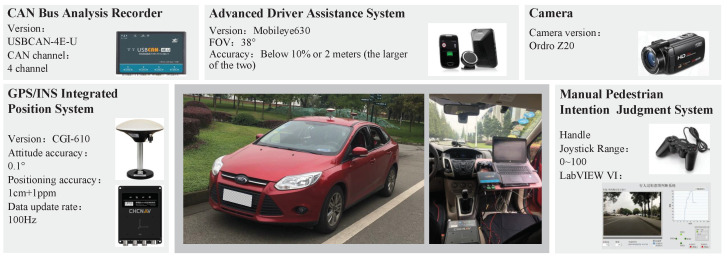
The test platform and its performance parameters.

A naturalistic driving test platform was established to collect data on vehicle kinematics and vehicle surroundings, involving pedestrians’ behaviour. The test vehicle equipped with data acquisition devices and their performance parameters are shown in Figure 1.

The Mobileye 630 used in this paper is a vision-based aftermarket ADAS, which includes forward collision warning (FCW), lane departure warning (LDW), and headway monitoring and warning (HMW) features. It can provide real-time location position relationships between vehicles, pedestrians, and lane lines. Vehicle kinematics data, involving speed, high-precision vehicle position, and acceleration, was acquired through a GPS and INS Integrated Positioning System. In addition to the Mobileye 630 camera, another camera was placed in front of the steering wheel, so that its view was consistent with the driver’s. This camera was used to record the pedestrians’ behaviour. Steering wheel angle, accelerator pedal position, and brake pedal trigger signal of the test vehicle were recorded by the CAN bus analysis recorder.

**Figure 2:**
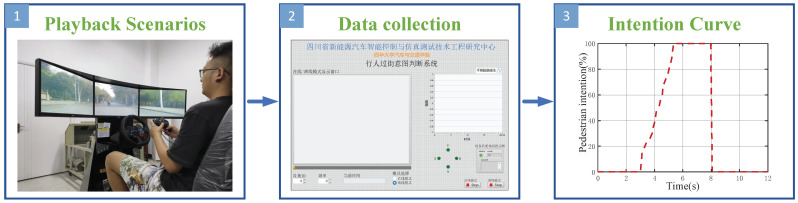
The manual pedestrian intention judgment system.

**Figure 3:**
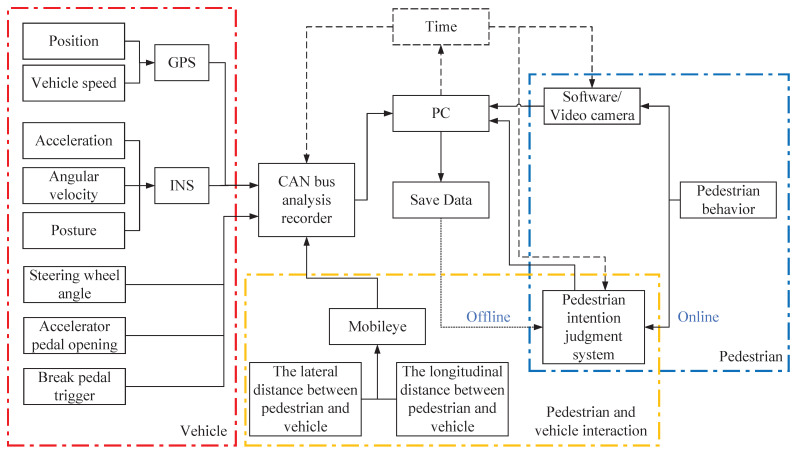
Block diagram of data collection.

In addition to the above objective data, the driver’s SJPCI was also needed in this study. A manual pedestrian intention judgment system was developed to collect the quantitative data of the SJPCI at every moment (Figure 2), which is different from the previous study that only considered whether pedestrians cross the street. With simple manipulation of the joystick, the real-time SJPCI can be recorded in the form of percentages. When it is judged that the pedestrian has no intention to cross the street, the joystick is placed in the initial position, i.e., the SJPCI is 0%; when it is judged that the pedestrian has crossing behaviour, the joystick is placed in the rightmost position, i.e., the SJPCI is 100%; the other different positions represent the intention intensity of pedestrians to cross the street. The data of SJPCI in the whole process of interaction change continuously over time. The SJPCI can be recorded online by the co-driver during the driving experiment process or offline by the driver during the videography playback process. In this paper, the offline videography data were used, as shown in Figure 2. Figure 3 shows the naturalistic driving data collection and output process. The test platform can keep the output of all objective and subjective data in sync.

### 2.3. Test Events

**Figure 4:**
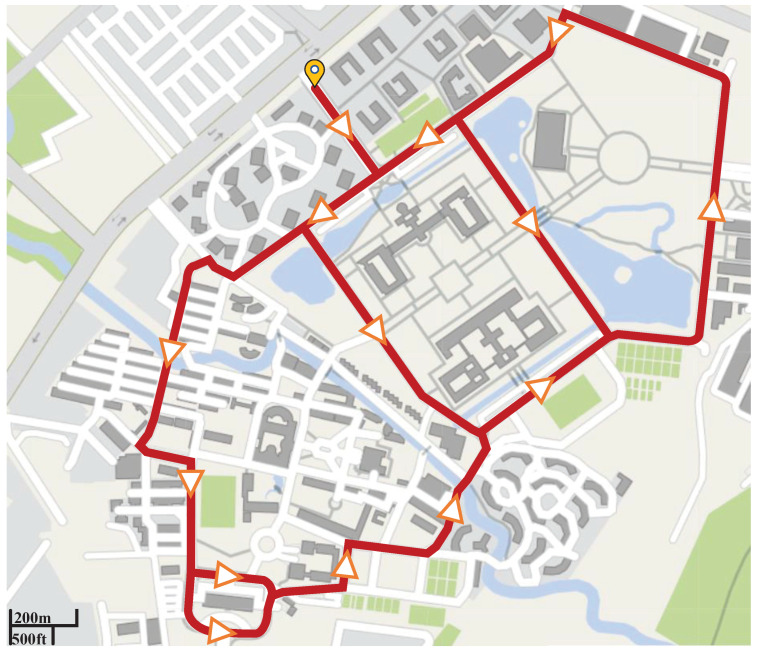
Map of the driving route.

The experiment was conducted on the streets of Xihua University campus. Figure 4 provides the map of the test route. All experiments were carried out during the day with good weather conditions from 9:00 a.m. to 5:00 p.m.

Each experiment was completed by three people, including the driver, co-driver, and safety instructor. The driver drove the test vehicle, the co-driver operated and controlled the manual pedestrian intention judgment system, and the rear safety instructor mainly provided safety reminders and informed the driver of the general driving route, but would not affect the driver’s driving behaviour and the co-driver’s judgment in any way. The driver was not informed of the purpose of the test and was asked to drive the vehicle according to their daily driving habits. Before the experiment, the driver was required to drive the test vehicle on the test route for half an hour to become familiar with the vehicle and its related operations.

The co-driver used the position of the joystick of the manual pedestrian intention judgment system to represent the pedestrians’ crossing intention. After the real vehicle test, the videos of pedestrians crossing the street were played back on the screen. The driver sat on the driving simulator, operated and controlled the joystick of the manual pedestrian intention judgment system to collect SJPCI, as shown in Figure 2. The pedestrians’ crossing intentions can change in real-time during pedestrian–vehicle interaction, so the entire real-time dynamic change process could be recorded. The results of co-driver’s SJPCI were not used in this paper, which will be carried out in a comparative study of SJPCI between the driver and co-driver in the future.

## 3. Results and Discussion

The pedestrian–vehicle interaction under natural driving conditions belongs to accidental and uncontrollable events. It needs to take a long experiment time to obtain a certain amount of data through testing. A total of 1274 pedestrian–vehicle interaction events with a total road mileage of about 980 km were collected.

During the pedestrian–vehicle interaction events, more than 70% of pedestrian movements were crossing incidents involving groups of two or more people [35], a behaviour that is also affected by pedestrians’ interaction with each other. Furthermore, different road conditions have an impact on pedestrian–vehicle interaction behaviour. Therefore, the screening of pedestrian–vehicle interaction events needs to meet the strict qualification of non-intersections, no traffic lights, no speed bumps, and single pedestrian crossing behaviour, to avoid their influence on the interaction behaviour. After the strict screening and processing of 1274 events, only 98 sets of valid pedestrian–vehicle interaction events were obtained. The 98 events were scattered along the whole driving route.

### 3.1. Data Overview and Statistics

Based on the 98 datasets of valid events, performance metrics including TTC, SJPCI, pedestrian position and crossing direction, and vehicle speed and deceleration were extracted. Moreover, the datasets of events were classified and analyzed according to the vehicle’s deceleration during the interaction. The purpose of classification based on the average vehicle deceleration is to effectively combine naturalistic decelerating behaviour with motion control in future AVs development to enable AVs to avoid pedestrians more naturally, safely, and smoothly.

Based on whether vehicle deceleration was triggered, the statistical data were divided into two types of typical scenarios: vehicle deceleration scenarios and non-deceleration scenarios. According to the changes in the average vehicle deceleration, the deceleration event conditions were classified into three types by the Ward system clustering method: mild deceleration, moderate deceleration, and severe deceleration (Table 1). The non-deceleration, mild deceleration, moderate deceleration, and severe deceleration events accounted for 38.8%, 8.2%, 36.7%, and 16.3% of interactions, respectively. These results were related to the characteristics of the campus test: two-way single-lane streets, relatively short crossing distance, and relatively low vehicle speed.

Table 1 shows the initial TTC at the beginning of pedestrian–vehicle interaction, the driver’s SJPCI, and the average vehicle deceleration in the entire pedestrian–vehicle interaction process. The initial TTC in non-deceleration scenarios represents the time when sensors detect pedestrians. The initial TTC in deceleration scenarios represents the initial time when the vehicle deceleration. SJPCI represents the percentage range of the interaction process. Since the data were clustered and analyzed according to the average deceleration, the TTC and SJPCI in the classification overlapped. The TTC ranged from 3.71 s to 7.71 s under the non-deceleration scenarios, and the distribution range of SJPCI was 0–100%. Under the mild deceleration scenarios, the TTC range at the start of deceleration was 3.20–6.28 s, the SJPCI ranged from 50% to 100%, and the average deceleration was −0.62–−0.23 m/s${}^{2}$. Under the moderate deceleration scenarios, the TTC range at the start of deceleration was 2.10–5.83 s, the distribution range of SJPCI was 80–100%, and the average deceleration was −1.48–−0.70 m/s${}^{2}$. The TTC range under the mild and moderate deceleration scenarios had a small difference. Under the severe deceleration scenarios, the TTC range at the start of deceleration was 1.68–3.86 s, the SJPCI was 100%, and the average deceleration was −3.08–−1.81 m/s${}^{2}$. From the overall trend, with the decrease in the TTC and the increase in SJPCI, the deceleration behaviour of vehicles was more significant. The scenarios’ classifications are reasonable and generalized based on the statistical data, but the detailed data may be subject to change with the test mileage.

Although the initial TTC measured in this paper was no more than 7.71 s in all events, this does not mean that the actual TTC at the beginning of the interaction between the vehicle and the pedestrian was less than 7.71 s. Due to the recognition capabilities of Mobileye and the uncontrollable random pedestrian crossing events, larger pedestrian–vehicle interaction TTC data had not been recorded in this round of tests. The influence of pedestrians on vehicles was almost negligible in the case of the larger TTC [6], so the data in this paper are sufficient to illustrate the influence of pedestrians crossing on driving behaviour.

In addition, different pedestrian crossing directions have an impact on the pedestrian–vehicle interaction, as shown in Table 2. The original data had a certain degree of dispersion, so a more intuitive average value was adopted for the statistical data. The average initial longitudinal distance and initial TTC when the driver took deceleration measures for pedestrians crossing from right to left are both less than those for pedestrians crossing from left to right. Pedestrians crossing from left to right accounted for 80% of the mild deceleration scenarios, and pedestrians crossing from left to right accounted for 70% of the moderate deceleration scenarios. In the severe deceleration scenarios, all the events were pedestrians crossing from right to left. This result is related to the fact that vehicles all drive on the right side of the roads in mainland China. The process of pedestrians crossing the entire street from left to right will affect the driver, but when pedestrians cross from right to left, the driver generally only needs to consider the impact of pedestrians on the lane where the vehicle is located.

### 3.2. Discussion on Different Scenarios

#### 3.2.1. Non-Deceleration Scenario

The non-deceleration scenario refers to the fact that the driver does not take any deceleration measures when encountering a pedestrian crossing event. The non-deceleration scenario mainly included two types: one was that pedestrians had the intention to cross the street and the behaviour of crossing the street, but driving safety was not affected due to the large TTC, so the driver did not take any deceleration measures; the other was that pedestrians had a clear no-crossing or low crossing intention, and the driver believed that the driving safety could be guaranteed without slowing down, even if the TTC was small. All the pedestrians had crossed the streets in the first case of the non-deceleration events. The TTC range was 4.47–7.71 s, and the SIPCI was 70–100%. In the second case, all the pedestrians ended up not crossing the street. The TTC range was 3.71–5.52 s, and the distribution range of SJPCI was 0–50%.

**Figure 5:**
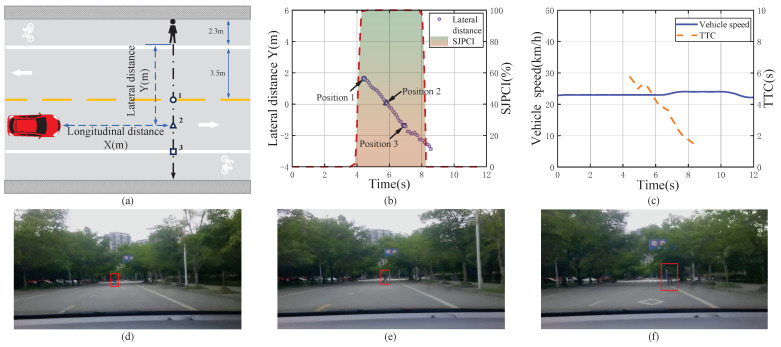
A non-deceleration event when encountering a pedestrian with a clear crossing intention and the data analysis results. (**a**) Pedestrian–vehicle interaction schematic diagram; (**b**) Lateral distance and SJPCI; (**c**) Vehicle speed and TTC; (**d**) Position 1: Pedestrians walked near the center line; (**e**) Position 2: Pedestrians walked between the center line and right lane; (**f**) Position 3: Pedestrians crossed the right lane line.

Figure 5 shows a non-deceleration event when encountering a pedestrian with a clear crossing intention and data analysis results. When the vehicle was travelling at about 23 km/h and encountered a pedestrian crossing the street at position 1 (near the centerline), the TTC was 5.63 s (Figure 5c), and the driver’s SJPCI was 100% based on the pedestrian’s behaviour (Figure 5b). Although the driver judged that the pedestrian’s crossing intention continued to be 100% in the interaction process, the initial value of the TTC was relatively large, i.e., the initial longitudinal distance between the vehicle and the pedestrian was long (36 m) and the vehicle speed was slow (23 km/h). Therefore, the driver believed that the pedestrian’s crossing behaviour would not affect driving safety in this case, and no deceleration measures were taken. The results illustrate that when the TTC is relatively large, the distance is long, and the speed is low, even if the pedestrian has a 100% intention to cross the street, the driver still tends to maintain a stable speed for observation.

**Figure 6:**
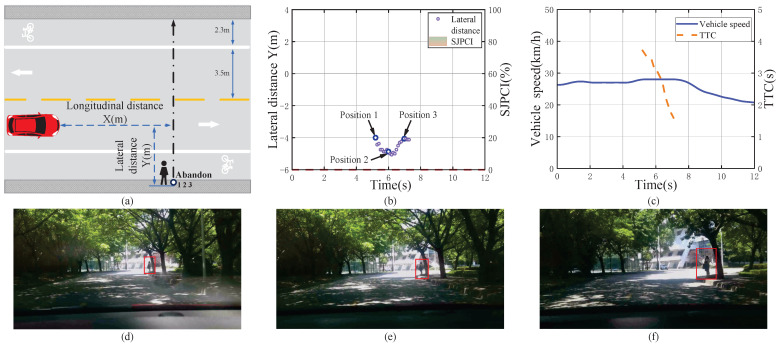
A non-deceleration event when encountering a pedestrian with a clear no-crossing intention and the data analysis results. (**a**) Pedestrian–vehicle interaction schematic diagram; (**b**) Lateral distance and SJPCI; (**c**) Vehicle speed and TTC; (**d**) Position 1: Pedestrians stood on the right shoulder; (**e**) Position 2: Pedestrians watched the vehicle; (**f**) Position 3: Pedestrians gave up crossing the street.

Another non-deceleration event when encountering a pedestrian with a clear no-crossing intention and data analysis results are shown in Figure 6. When the vehicle was travelling at about 27 km/h and encountered a pedestrian standing on the right shoulder (position 1) in front, the TTC was 3.71 s. At this time, the driver judged that the pedestrian’s crossing intention was 0% from the behaviour that the pedestrian was making a phone call. Since the pedestrian was always far away from the street, the driver’s SJPCI continued to be 0% in the interaction process. The driver believed that the pedestrian would give up crossing and not affect driving safety in this situation, so no obvious deceleration measures were taken, and the driver basically kept driving at a medium speed fluctuating normally from 27 km/h to 28 km/h during the interaction process. The results show that when the pedestrian crossing intention is 0%, the driver chooses to drive at a stable speed to observe the pedestrian’s behaviour, even if the TTC is relatively small.

#### 3.2.2. Deceleration Scenario

The deceleration scenario refers to the deceleration measures taken when the vehicle encounters a pedestrian crossing event, including mild deceleration, moderate deceleration and severe deceleration. It is analyzed from the existing data statistics (Table 1) that when 3.2 s < TTC < 6.28 s, the vehicle showed mild deceleration behavior with an average deceleration of −0.62–−0.23 m/s${}^{2}$; when 2.10 s < TTC < 5.83 s, the vehicle provided a moderate deceleration behavior with an average deceleration of −1.48–−0.70 m/s${}^{2}$; and when 1.68 s < TTC < 3.86 s, the vehicle showed a severe deceleration behavior with an average deceleration of −3.08–−1.81 m/s${}^{2}$.

##### Mild Deceleration

**Figure 7:**
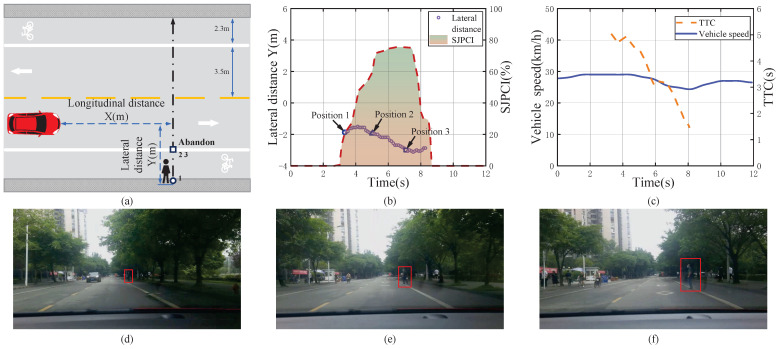
A mild deceleration event when encountering a pedestrian crossing the street and the data analysis results. (**a**) Pedestrian–vehicle interaction schematic diagram; (**b**) Lateral distance and SJPCI; (**c**) Vehicle speed and TTC; (**d**) Position 1: Pedestrians started crossing from the right lane line; (**e**) Position 2: Pedestrians crossed the right lane line; (**f**) Position 3: Pedestrians gave up crossing the street.

Figure 7 shows a mild deceleration event when encountering a pedestrian crossing the street and the data analysis results. The vehicle encountered a pedestrian who might cross the street when it was travelling at about 29 km/h. The initial longitudinal distance between the vehicle and the pedestrian was long (40 m), and the vehicle speed was medium (29 km/h). Based on the pedestrian’s behaviour, the driver judged that the pedestrian’s crossing intention gradually increased to about 75%. Meanwhile, the pedestrian had not noticed the test vehicle. The driver took mild braking measures to decelerate the test vehicle, and the TTC was 5.01 s at this time. Then, the pedestrian suddenly turned his head to observe the rear and noticed the vehicle. The pedestrian gave up crossing and chose to give way. The driver’s SJPCI was reduced to 30%, and they began to accelerate. The vehicle speed changed from 29 km/h to 24 km/h during the pedestrian–vehicle interaction, and the average deceleration was about −0.28 m/s${}^{2}$. Therefore, the results indicate that the driver chose to avoid the pedestrian with a mild deceleration in this scenario when the TTC was relatively large, the initial vehicle speed was relatively high, and the pedestrian had a high crossing intention.

##### Moderate Deceleration

**Figure 8:**
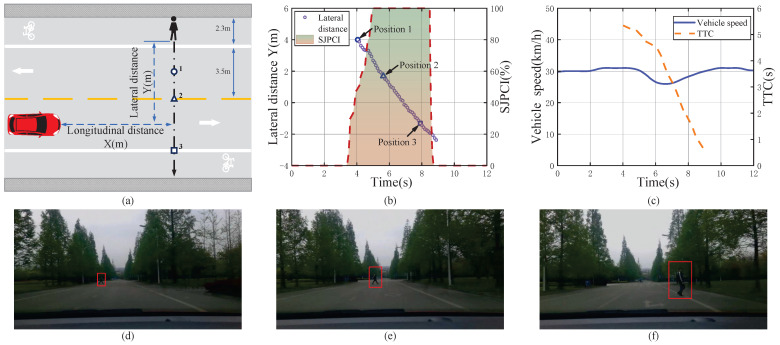
A moderate deceleration event when encountering a pedestrian crossing the street and the data analysis results. (**a**) Pedestrian–vehicle interaction schematic diagram; (**b**) Lateral distance and SJPCI; (**c**) Vehicle speed and TTC; (**d**) Position 1: Pedestrians started crossing the left lane line; (**e**) Position 2: Pedestrians walked to the center line; (**f**) Position 3: Pedestrians crossed the right lane line.

A moderate deceleration event when encountering a pedestrian crossing the street and the data analysis results are shown in Figure 8. When the vehicle was travelling at a speed of about 31 km/h, the driver found that a pedestrian might cross the street from left to right. According to the pedestrian’s behaviour, it was judged that the pedestrian’s intention to cross the street rapidly increased from 0% to 100%, and then the pedestrian made a clear crossing behaviour at a fast walking pace. Although the TTC was large at 5.33 s at this time, the driver’s SJPCI continued to be 100%, and he believed that the pedestrian’s crossing behaviour affected driving safety in this situation, so moderate deceleration measures were taken. The vehicle speed changed from 31 km/h to 26 km/h during the vehicle–pedestrian interaction, and the average deceleration was −1.13 m/s${}^{2}$. Although the TTC at the time of deceleration triggered in this moderate deceleration event was larger than that in the above mild deceleration event, the SJPCI and vehicle speed were higher, which posed a greater threat to the driver’s driving safety, resulting in greater braking deceleration. That is, the driver tends to avoid pedestrians with moderate deceleration behaviour in the scenario of large TTC, high SJPCI, and high vehicle speed.

##### Severe Deceleration

**Figure 9:**
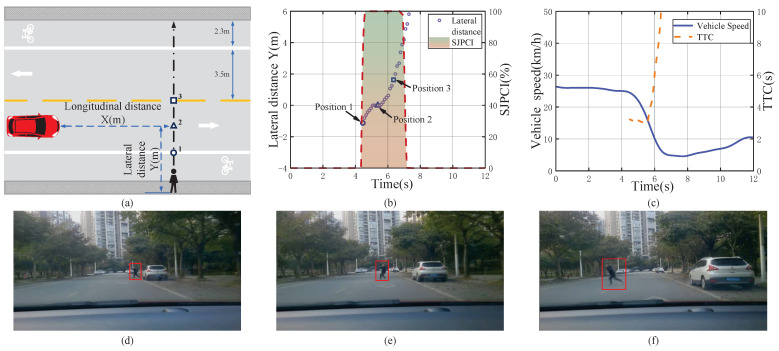
A severe deceleration event when encountering a pedestrian crossing the street and the data analysis results. (**a**) Pedestrian–vehicle interaction schematic diagram; (**b**) Lateral distance and SJPCI; (**c**) Vehicle speed and TTC; (**d**) Position 1: Pedestrian suddenly appeared from behind a vehicle; (**e**) Position 2: Pedestrian had seen the approaching vehicle; (**f**) Position 3: Pedestrian ran to cross the street.

Figure 9 shows a severe deceleration event when encountering a pedestrian crossing the street and the data analysis results. When travelling at about 26 km/h, a vehicle encountered a pedestrian who suddenly appeared from the side of the vehicle parked at the roadside, and the driver’s SJPCI was sharply increased to 100% based on his behaviour. The initial distance between the vehicle and the pedestrian was relatively short (20 m) and the vehicle speed was medium at 25 km/h. At this time, the TTC value was small at 3.24 s, and the driver’s SJPCI continued to be 100%. The driver believed that the crossing behaviour seriously affected driving safety, so they adopted severe braking behaviour. Then, the pedestrian suddenly turned his head and noticed the vehicle. The pedestrian stopped crossing the street, but at this time, the pedestrian was already at position 2. The driver continued to brake due to the high SJPCI and short distance. After the pedestrian realized that the vehicle braked to yield, he continued to cross the street. The vehicle speed changed from 25 km/h to 5 km/h during the pedestrian–vehicle interaction, with an average deceleration at about −3.08 m/s${}^{2}$. The results indicate that the driver tends to avoid pedestrians with a severe deceleration in the scenario of small TTC, high SJPCI, and short distance.

## 4. Conclusions

In this paper, NDS was used to study the underlying behavioural mechanisms of how vehicles avoid pedestrians. A manual pedestrian intention judgment system was first developed, and the quantitative data of SJPCI at every moment throughout the interaction process was first collected and analyzed. In total, 1274 pedestrian–vehicle interaction events were collected. From these, 98 single-pedestrian crossing events of interest were screened and classified by the Ward system clustering method into the non-deceleration scenario, mild deceleration scenario, moderate deceleration scenario, and severe deceleration scenario, accounting for 38.8%, 8.2%, 36.7%, and 16.3% of events, respectively.

This study provides data statistics and process analyses of vehicle deceleration behaviour in different interaction scenarios. The results show that the vehicle deceleration behaviour is relative to initial TTC, SJPCI, vehicle speed, pedestrian position and crossing direction. With the quantitative data and different scenarios classification, human-like AVs can be developed to avoid pedestrians more naturally, safely, and smoothly.

## Figures and Tables

**Table 1:** Data statistics of different scenarios.

Scenarios		Initial TTC (s)	SJPCI	Average Deceleration (m/s${}^{2}$)
Non-deceleration scenarios		3.71–7.71	0–100%	0
Deceleration scenarios	Mild deceleration	3.20–6.28	50–100%	−0.62–−0.23
	Moderate deceleration	2.10–5.83	80–100%	−1.48–−0.70
	Severe deceleration	1.68–3.86	100%	−3.08–−1.81

**Table 2:** The impact of different crossing directions on vehicle behaviour.

Scenarios	Crossing Direction	Average Initial TTC (s)	Average Initial Distance (m)
Mild deceleration	Left→right	5.57	35.70
	Right→left	5.27	32.67
Moderate deceleration	Left→right	3.89	33.01
	Right→left	3.74	29.41
Severe deceleration	Right→left	2.24	16.78

## Data Availability

The datasets in this study are available from the corresponding author on reasonable request.

## References

[1] Rasouli A., Tsotsos J.K. (2020). Autonomous Vehicles That Interact With Pedestrians: A Survey of Theory and Practice. IEEE Trans. Intell. Transp. Syst..

[2] Schmitt P., Britten N., Jeong J., Coffey A., Clark K., Kothawade S.S., Grigore E.C., Khaw A., Konopka C., Pham L. (2022). Can Cars Gesture? A Case for Expressive Behavior Within Autonomous Vehicle and Pedestrian Interactions. IEEE Robot. Autom. Lett..

[3] Camara F., Dickinson P., Fox C. (2021). Evaluating pedestrian interaction preferences with a game theoretic autonomous vehicle in virtual reality. Transp. Res. Part F Traffic Psychol. Behav..

[4] Prédhumeau M., Mancheva L., Dugdale J., Spalanzani A. (2022). Agent-Based Modeling for Predicting Pedestrian Trajectories around an Autonomous Vehicle. J. Artif. Intell. Res..

[5] Jayaraman S.K., Tilbury D.M., Yang X.J., Pradhan A.K., Robert L.P. Analysis and Prediction of Pedestrian Crosswalk Behavior during Automated Vehicle Interactions. Proceedings of the 2020 IEEE International Conference on Robotics and Automation (ICRA).

[6] Schneemann F., Gohl I. Analyzing driver-pedestrian interaction at crosswalks: A contribution to autonomous driving in urban environments. Proceedings of the 2016 IEEE Intelligent Vehicles Symposium (IV).

[7] Koilias A., Mousas C., Rekabdar B. The Effects of Driving Habits on Virtual Reality Car Passenger Anxiety. Proceedings of the Virtual Reality and Augmented Reality.

[8] Koilias A., Mousas C., Rekabdar B., Anagnostopoulos C.N. Passenger Anxiety when Seated in a Virtual Reality Self-Driving Car. Proceedings of the 2019 IEEE Conference on Virtual Reality and 3D User Interfaces (VR).

[9] Dalipi A.F., Liu D., Guo X., Chen Y., Mousas C. VR-PAVIB: The Virtual Reality Pedestrian-Autonomous Vehicle Interaction Benchmark. Proceedings of the 12th International Conference on Automotive User Interfaces and Interactive Vehicular Applications.

[10] Schmitt P., Britten N., Jeong J., Coffey A., Clark K., Kothawade S.S., Grigore E.C., Khaw A., Konopka C., Pham L. (2022). nuReality: A VR environment for research of pedestrian and autonomous vehicle interactions. arXiv.

[11] Che X., Li C., Zhang Z. A Test Method for Self-driving Vehicle Based on Mixed Reality. Proceedings of the 2021 IEEE International Conference on Smart Internet of Things (SmartIoT).

[12] Yang L., Wang R., Zhao X., Xu Z., Yang Y. (2021). CAVTest: A Closed Connected and Automated Vehicles Test Field of Chang’an University in China. SAE Int. J. Connect. Autom. Veh..

[13] Tian R., Li L., Yang K., Chien S., Chen Y., Sherony R. Estimation of the vehicle-pedestrian encounter/conflict risk on the road based on TASI 110-car naturalistic driving data collection. Proceedings of the 2014 IEEE Intelligent Vehicles Symposium Proceedings.

[14] Bai H., Cai S., Ye N., Hsu D., Lee W.S. Intention-aware online POMDP planning for autonomous driving in a crowd. Proceedings of the 2015 IEEE International Conference on Robotics and Automation (ICRA).

[15] Rasch A., Panero G., Boda C.-N., Dozza M. (2020). How do drivers overtake pedestrians? Evidence from field test and naturalistic driving data. Accid. Anal. Prev..

[16] Barnard Y., Utesch F., van Nes N., Eenink R., Baumann M. (2016). The study design of UDRIVE: The naturalistic driving study across Europe for cars, trucks and scooters. Eur. Transp. Res. Rev..

[17] Wang T., Wu J., McDonald M. A micro-simulation model of pedestrian–vehicle interaction behavior at unsignalized mid-block locations. Proceedings of the 2012 15th International IEEE Conference on Intelligent Transportation Systems.

[18] Zhu J., Chen S., Tu W., Sun K. (2019). Tracking and Simulating Pedestrian Movements at Intersections Using Unmanned Aerial Vehicles. Remote. Sens..

[19] Sheykhfard A., Haghighi F., Papadimitriou E., Van Gelder P. (2021). Analysis of the occurrence and severity of vehicle-pedestrian conflicts in marked and unmarked crosswalks through naturalistic driving study. Transp. Res. Part F Traffic Psychol. Behav..

[20] Lyu N., Deng C., Xie L., Wu C., Duan Z. (2019). A field operational test in China: Exploring the effect of an advanced driver assistance system on driving performance and braking behavior. Transp. Res. Part F Traffic Psychol. Behav..

[21] Hariyono J., Jo K.H. Detection of pedestrian crossing road. Proceedings of the 2015 IEEE International Conference on Image Processing (ICIP).

[22] Varytimidis D., Alonso-Fernandez F., Duran B., Englund C. Action and Intention Recognition of Pedestrians in Urban Traffic. Proceedings of the 2018 14th International Conference on Signal-Image Technology & Internet-Based Systems (SITIS).

[23] Chaabane M., Trabelsi A., Blanchard N., Beveridge R. Looking ahead: Anticipating pedestrians crossing with future frames prediction. Proceedings of the IEEE/CVF Winter Conference on Applications of Computer Vision.

[24] Sucha M., Dostal D., Risser R. (2017). Pedestrian-driver communication and decision strategies at marked crossings. Accid. Anal. Prev..

[25] Fu T., Miranda-Moreno L., Saunier N. (2018). A novel framework to evaluate pedestrian safety at non-signalized locations. Accid. Anal. Prev..

[26] Yang W., Zhang X., Lei Q., Cheng X. (2019). Research on Longitudinal Active Collision Avoidance of Autonomous Emergency Braking Pedestrian System (AEB-P). Sensors.

[27] Kathuria A., Vedagiri P. (2020). Evaluating pedestrian vehicle interaction dynamics at un-signalized intersections: A proactive approach for safety analysis. Accid. Anal. Prev..

[28] Sheykhfard A., Haghighi F. (2019). Performance analysis of urban drivers encountering pedestrian. Transp. Res. Part F Traffic Psychol. Behav..

[29] Völz B., Behrendt K., Mielenz H., Gilitschenski I., Siegwart R., Nieto J. A data-driven approach for pedestrian intention estimation. Proceedings of the 2016 IEEE 19th International Conference on Intelligent Transportation Systems (ITSC).

[30] Keller C.G., Hermes C., Gavrila D.M. (2011). Will the Pedestrian Cross? Probabilistic Path Prediction Based on Learned Motion Features. Proceedings of the Pattern Recognition.

[31] Gorrini A., Crociani L., Vizzari G., Bandini S. (2018). Observation results on pedestrian–vehicle interactions at non-signalized intersections towards simulation. Transp. Res. Part F Traffic Psychol. Behav..

[32] Fu T., Hu W., Miranda-Moreno L., Saunier N. (2019). Investigating secondary pedestrian–vehicle interactions at non-signalized intersections using vision-based trajectory data. Transp. Res. Part C Emerg. Technol..

[33] Jiang C., Qiu R., Fu T., Fu L., Xiong B., Lu Z. (2020). Impact of right-turn channelization on pedestrian safety at signalized intersections. Accid. Anal. Prev..

[34] Hu L., Ou J., Huang J., Wang F., Wang Y., Ren B., Peng H., Zhou L. (2021). Safety evaluation of pedestrian–vehicle interaction at signalized intersections in Changsha, China. J. Transp. Saf. Secur..

[35] Rudenko A., Palmieri L., Lilienthal A.J., Arras K.O. Human Motion Prediction Under Social Grouping Constraints. Proceedings of the 2018 IEEE/RSJ International Conference on Intelligent Robots and Systems (IROS).

